# Characterization of Aroma Composition of *Amomum tsaoko* During the Drying Process Based on GC–MS

**DOI:** 10.1002/fsn3.4726

**Published:** 2025-01-09

**Authors:** Hui Wen, Meiquan Yang, Zongliang Xu, Tianmei Yang, Jinyu Zhang

**Affiliations:** ^1^ Institute of Medicinal Plants Yunnan Academy of Agricultural Sciences Kunming China; ^2^ School of Agriculture Yunnan University Kunming China

**Keywords:** *Amomum tsaoko*, different drying temperatures, drying process, GC–MS, metabolites

## Abstract

*Amomum tsaoko* is an important spice and medicinal plant widely utilized in East and Southeast Asia. Non‐targeted metabolomics techniques were employed to study the variations in the content and composition of essential oil from *A. tsaoko* during drying at different temperatures: 40°C, 50°C, 60°C, and 70°C. A total of 260 metabolites were detected using gas chromatography–mass spectrometry (GC–MS), mainly terpenoids and aldehydes. Cineole, the most important component, accumulated abundantly in samples dried at 50°C. A higher temperature (70°C) was conducive to the accumulation of aldehydes. Overall, the optimal drying condition for *A. tsaoko* was determined to be 50°C for 50 h. In addition, nine differential metabolites were screened using variable important in projection and *p* value (VIP > 1 and *p* < 0.05), which may serve as potential flavor markers to differentiate various drying treatments of *A. tsaoko* . This study provides a novel perspective on understanding the dynamic metabolites changes during the drying process, and establishes a theoretical foundation for the refinement and high‐quality processing of *A. tsaoko* .

## Graphical abstract

This study measured the morphological data, moisture content, oil yield, enzyme activity, and metabolite changes of *Amomum tsaoko* at different drying temperatures and times. The results showed that the optimal drying temperature and time were 50°C for 50 h, furthermore, nine differential metabolites were screened, which can be used as potential flavor markers to distinguish different drying conditions. This study establishes a theoretical foundation for the refinement and high‐quality processing of *A. tsaoko* .

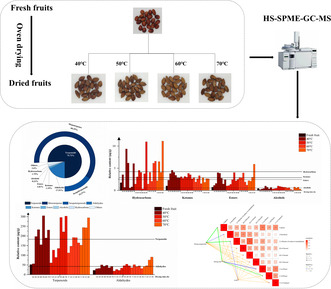

## 1 Introduction

*Amomum tsaoko* Crevost & Lemarié is a perennial herbaceous plant belonging to the Zingiberaceae family, predominantly found in southwestern China and northern Vietnam (He et al. 2020). The dried and mature fruits exhibit extensive edible and medicinal value (Liu et al. 2018). Chemical studies have shown that the primary active compounds are essential oils and flavonoids, which possess pharmacological effects such as gastrointestinal regulation, antibacterial activity, and blood sugar reduction (Cui et al. 2017). At present, more than 300 compounds have been detected in *A. tsaoko* , of which at least 209 compounds have been isolated and identified (Yang et al. 2022). Due to its potent aroma, the essential oil of *A. tsaoko* has garnered widespread attention. The essential oil primarily consists of terpenoids, aldehydes, alkanes, and other components. Liu et al. (2023) employed steam distillation to extract essential oils from *A. tsaoko* , resulting in the identification of 34 metabolites. Additionally, they utilized an ultrasound‐assisted organic solvent method for extraction, which led to the identification of 43 metabolites. Liang et al. (2023) analyzed fresh fruits of *A. tsaoko* sourced from four production areas and identified 50 compounds. Among these, the primary volatile compounds were (*E*)‐2‐octenal, (*E*)‐2‐decenal, 2‐isopropylbenzaldehyde, neral, geranial, and eucalyptol.

The physical and chemical properties of aromatic plants are determined by their moisture content (Rocha, Melo, and Radunz 2011). Due to their high moisture content, fresh fruits of *A. tsaoko* are prone to decay and deterioration. Drying is the primary method for preserving the quality of aromatic and medicinal plants, with the aim of preventing enzyme and microbial activity, thereby extending their shelf life (Qin et al. 2022). Natural drying of *A. tsaoko* results in a higher incidence of mold, making artificial drying the preferred method (Thamkaew, Sjöholm, and Galindo 2020). Various factors such as drying methods and temperatures can have a certain impact on the essential oil composition and biological activity of *A. tsaoko* . Qin et al. (2021) studied the effects of 12 different pre‐drying and drying methods on the chemical composition of *A. tsaoko* , among which the combination of oven drying and sun drying was the optimal drying method. Wang et al. (2021) employed metabolomics to analyze the essential oil composition and content of *A. tsaoko* at various temperatures (25°C, 40°C, 55°C, 70°C, 85°C, and 100°C). They found that the sample dried at 55°C had the highest oil yielded, while the sample dried at 100°C had the highest eucalyptol content. However, these studies focused solely on drying methods or temperatures, without determining the optimal drying temperature and duration for *A. tsaoko* . In order to quickly dry fruits in the market, increasing the drying temperature to shorten the drying time can alter the fruit's metabolic components and lead to the loss of active ingredients, diminishing its medicinal and culinary value. Therefore, investigating the optimal drying temperature and time is crucial for guiding the processing of *A. tsaoko* . Reports exist on metabolite changes in other species at different temperatures and time, such as *Trachinotus ovatus* (Qiu et al. 2023), *Flammulina velutipes* (Yang et al. 2016), *Coffea canephora* (Zhang et al. 2022), *Camellia sinensis* (Wang et al. 2023), *Ziziphus jujuba* (Gou et al. 2023), and *Gardenia jasminoides* (Yu et al. 2023). During the drying process, volatile compounds have a significant impact on the final product, and the temperature sensitivity between different species varies (Rocha, Melo, and Radunz 2011). However, there are currently no reports on the changes in volatile compounds of *A. tsaoko* at different temperatures and time. Therefore, elucidating the compound changes of *A. tsaoko* during the drying process is essential for standardizing the quality of the final product.

Relying solely on basic aroma evaluation is insufficient to solve the mystery of aroma formation. Metabolomics is an important tool for profiling compounds within analytes (Li et al. 2020). GC–MS is commonly used for the separation and identification of complex components, with advantages such as high sensitivity and fast analysis speed (Garcia and Barbas 2011). It is mainly used for food flavor analysis and has found widespread application in variety identification, origin certification, and pesticide residue detection (Ali et al. 2022; Rocchi et al. 2019; Yu et al. 2022). With the advancement of technology, more research is combining GC–MS with other extraction or analysis methods. For example, Xu et al. (2022) used GC–MS combined with an olfactory port to analyze odor active substances in French fries.

In order to better analyze the volatile flavor components in *A. tsaoko* , this study used headspace solid‐phase microextraction (HS‐SPME) combined with GC–MS technology to study the evolution and formation of volatile compounds during oven‐drying at different temperatures and time. Key volatile compounds influencing flavor changes during the drying process were identified. This study aims to establish a theoretical foundation for understanding the formation mechanism of flavor substances in *A. tsaoko* during drying, and the research results can provide reference guidance for producing high‐quality *A. tsaoko* and enhancing its flavor quality.

## 2 Materials and Methods

### 2.1 Plant Materials

Approximately 60 kg of fresh and mature fruits were harvested at the Nujiang Plantation in Yunnan. All fruits were identified by Dr. Jinyu Zhang (Medicinal Plants Research Institute, Yunnan Academy of Agricultural Sciences, Kunming, China) as the fruits of *A. tsaoko* in Zingiberaceae.

The fresh and ripe fruits collected directly from local plantations were randomly divided into four groups and were dried using an electric thermostatic blast oven at the temperatures of 40°C, 50°C, 60°C, and 70°C, respectively. Samples were taken at different drying time points of each temperature: 40°C (10, 20, 30, 40, 50, 60, 70 h), 50°C (5, 10, 15, 20, 30, 40, 50 h), 60°C (5, 10, 15, 20, 30), and 70°C (5, 10, 15, 20 h), along with a fresh fruit sample. A total of 24 groups of samples with different treatments.

### 2.2 Measurement of Quality Indicators

Firstly, the morphological data of the samples were measured at different drying temperatures and time, including fruit length, fruit width, fruit shell thickness, fruit weight, fruit shell weight, and seed cluster weight. Thirty fruits from each stage were selected for measurement.

Then, a moisture meter was used to measure the moisture content of the fruit samples at different drying temperatures and time. The moisture meter was set to 105°C and the timing was set to automatic. Three biological replicates were performed for each treatment condition.

### 2.3 Extraction of Volatile Oil

The extraction of volatile oil was carried out according to method A for the determination of volatile oil in the Pharmacopeia of the People's Republic of China (2020) (General Rule 2204). The peels of samples were peeled off, and the seed clusters were taken. After that, the seed clusters were ground into a fine powder and passed a 50‐mesh sieve. The essential oil was extracted by steam distillation, where 20.0 g of fine powder was accurately weighed and placed in a 500 mL round‐bottom flask with 300 mL of pure water. The mixture was kept faintly boiling until the amount of essential oil was no longer increased. The extraction time (5 h) was counted from the start of the boiling process (Zhang et al. 2020). The volume of oil was divided by the weight of seed clusters to calculate the oil yield (v/w). Three biological replicates were performed for each treatment condition.

### 2.4 Determination of Key Enzyme Activity in Flavor Synthesis

The activity of key enzyme involved in flavor synthesis was measured in different treatment samples, including 1,8‐cineole cyclase (1,8‐CC) and lipoxygenase (LOX). Enzyme activities were measured using ELISA kits: 1,8‐cineole cyclase (Item Number: MM‐6352501) and lipoxygenase (Item Number: MM‐3609901) (Hanáková et al. 2017).

### 2.5 GC–MS Conditions

The sample was pretreated by solid‐phase microextraction (SPME) technology and used a CTC three‐in‐one automatic sampler with an extraction head (50/30 μm DVB/CAR on PDMS). It was oscillated at 250 rpm for 15 min and then extracted for 30 min at 50°C. The desorption time was 5 min, and the GC cycle time was 50 min. The 0.5 g of the sample was added to a 20 mL sealed headspace bottle and detected using a 7890B‐5977B gas chromatography‐mass spectrometer (Agilent Technologies Co. Ltd.) with a DB‐WAX column (30 m × 0.25 mm × 0.25 μm). Helium gas was used at a constant flow rate of 1 mL/min to separate derivative substances. The temperature program was as follows: the sample inlet temperature was 260°C, the initial temperature was set to 40°C and held for 5 min, the temperature was heated to 220°C at a rate of 5 °C/min, then heated to 250°C at a rate of 20 °C/min and held for 2.5 min. The interface temperature was set to 260°C, the ion source temperature was set to 230°C, and the quadrupole temperature was set to 150°C. The electron ionization (EI) mode was set to 70 EV, and the quality range of full scan mode was 20–400 m/z (Tunnisa et al. 2022). Three biological replicates were performed for each treatment condition.

### 2.6 Compound Identification and Quantification

The GC–MS data were compared with the standard mass spectrometry database (National Institute of Standards and Technology Library 2014) and the published literature to obtain compound information, retention time, and peak areas. To enable comparison data of varying magnitudes, peak areas were normalized using an internal standard. The relative content of each metabolite was calculated based on the concentration and peak area of the internal standard.

### 2.7 Statistical Analysis

The one‐way ANOVA analysis of data was performed on SPSS 18.0 software. The partial least squares discriminant analysis (PLS‐DA) of metabolic compounds was performed using SIMCA 14.1. The variable important in projection (VIP) scores of metabolites were performed on MetaboAnalyst 5.0. The Mantel test analysis was completed on the BioDeep Platform (https://www.biodeep.cn). Most images were drawn using Origin 2023.

## 3 Results and Discussion

### 3.1 Analysis of Quality Indicator

**FIGURE 1:**
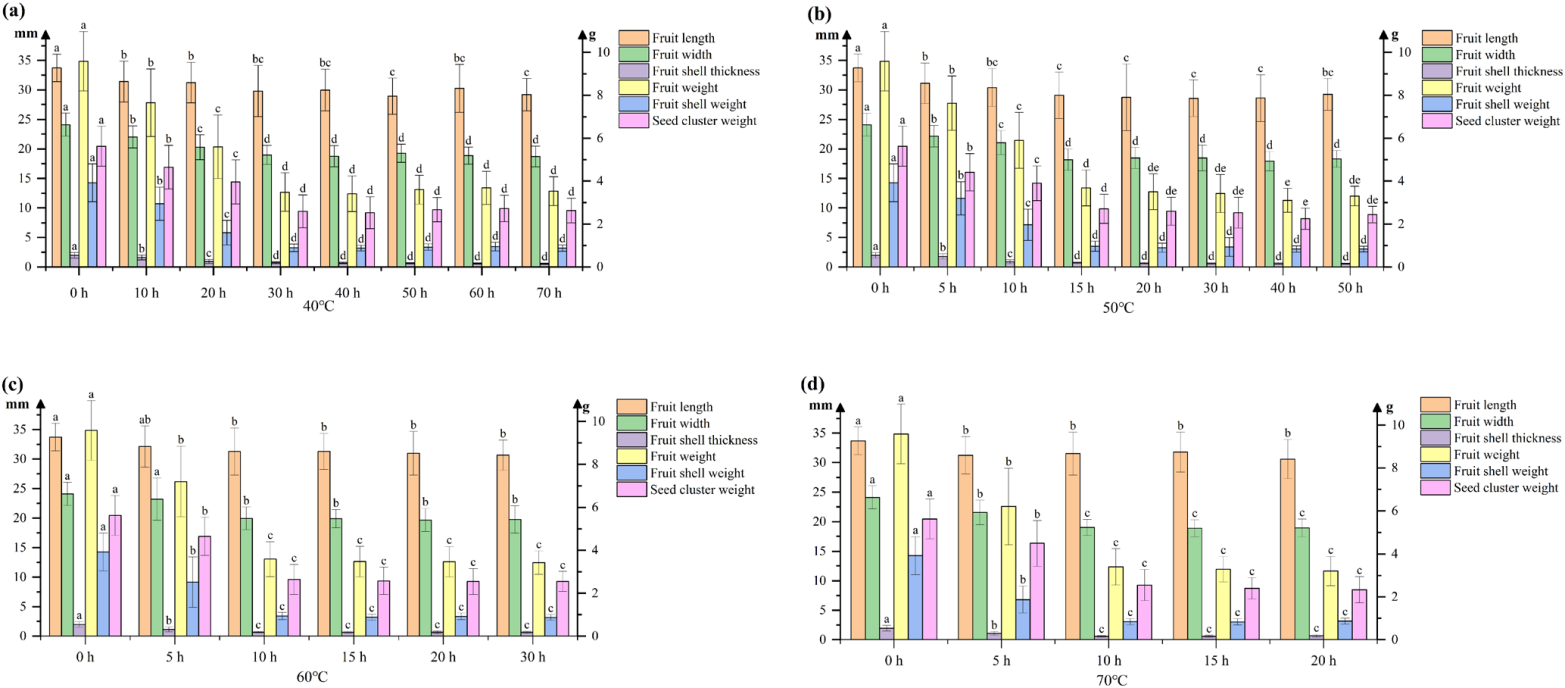
Changes in morphological data of *A. tsaoko* During Drying at Four Temperature Gradients of 40°C (a), 50°C (b), 60°C (c), and 70°C (d).

Morphological changes in *A. tsaoko* during the drying process were measured at four temperature gradients: 40°C, 50°C, 60°C and 70°C, as shown in Figure 1. The results show that the changes of morphological data at the initial stage of drying are the most significant, specifically within the 0–30 h at 40°C, 0–15 h at 50°C, 0–10 h at 60°C, and 0–10 h at 70°C.

The effect of different drying temperatures and time on the moisture content and oil yield of *A. tsaoko* is shown in Table 1. As the drying temperature increases, the rate of moisture content reduction in *A. tsaoko* accelerates. The slower moisture reduction rates observed during the early stages of drying at 40°C and 50°C are attributed to the high moisture content of the fresh fruits. In the early stage, a large amount of moisture evaporates from the fruits, and the lower temperatures cause moisture accumulation in the oven, resulting in a decreased evaporation rate. This stage is particularly susceptible to mold growth during the drying process. The Pharmacopoeia of the People's Republic of China (2020) stipulates that the moisture content of *A. tsaoko* shall not exceed 15.0%. Drying at 40°C for 60 h, 50°C for 50 h, 60°C for 20 h, and 70°C for 20 h all meet the specified standards.

**TABLE 1:** Data on moisture content and oil yield during different drying temperatures.

Drying temperature (°C)	Drying time (h)	Moisture content^a^ (%)	Oil yield^a^ (%)
40	0	69.28 ± 1.65	1.62 ± 0.0008
	10	69.00 ± 0.95	1.63 ± 0.0013
	20	59.18 ± 3.39	1.77 ± 0.0018
	30	41.64 ± 0.78	2.18 ± 0.0019
	40	33.81 ± 1.55	2.22 ± 0.0008
	50	21.44 ± 1.20	3.00 ± 0
	60	10.63 ± 0.55	3.35 ± 0.0022
	70	5.35 ± 0.11	2.40 ± 0
50	5	66.82 ± 1.23	1.57 ± 0.0021
	10	64.20 ± 0.63	1.67 ± 0.0014
	15	60.00 ± 3.38	2.67 ± 0.0043
	20	53.47 ± 3.00	3.00 ± 0.0010
	30	25.95 ± 2.45	3.03 ± 0.0024
	40	16.97 ± 1.82	2.50 ± 0.0025
	50	7.14 ± 1.03	2.08 ± 0.0014
60	5	55.90 ± 1.55	1.75 ± 0.0013
	10	37.85 ± 2.26	2.50 ± 0
	15	22.52 ± 1.41	2.57 ± 0.0015
	20	9.50 ± 0.13	3.07 ± 0.0006
	30	3.92 ± 0.49	2.97 ± 0.0006
70	5	47.90 ± 0.73	1.82 ± 0.0010
	10	36.73 ± 3.16	2.50 ± 0.0009
	15	15.43 ± 0.53	2.48 ± 0.0003
	20	4.48 ± 0.48	2.43 ± 0.0012

^a^ The moisture content and oil yield were expressed as the average ± standard deviation of three measurements.

The oil yield of *A. tsaoko* shows a trend of increasing firstly and then decreasing with prolonged drying time at the same temperatures. The highest oil yields are achieved after drying at 40°C for 60 h (3.35%), 50°C for 30 h (3.03%), 60°C for 20 h (3.07%), and 70°C for 10 h (2.50%). The optimal drying condition for achieving the specified moisture content and the highest oil yield of *A. tsaoko* is drying at 40°C for 60 h.

### 3.2 Analysis of Flavor Enzyme Activity

**FIGURE 2:**
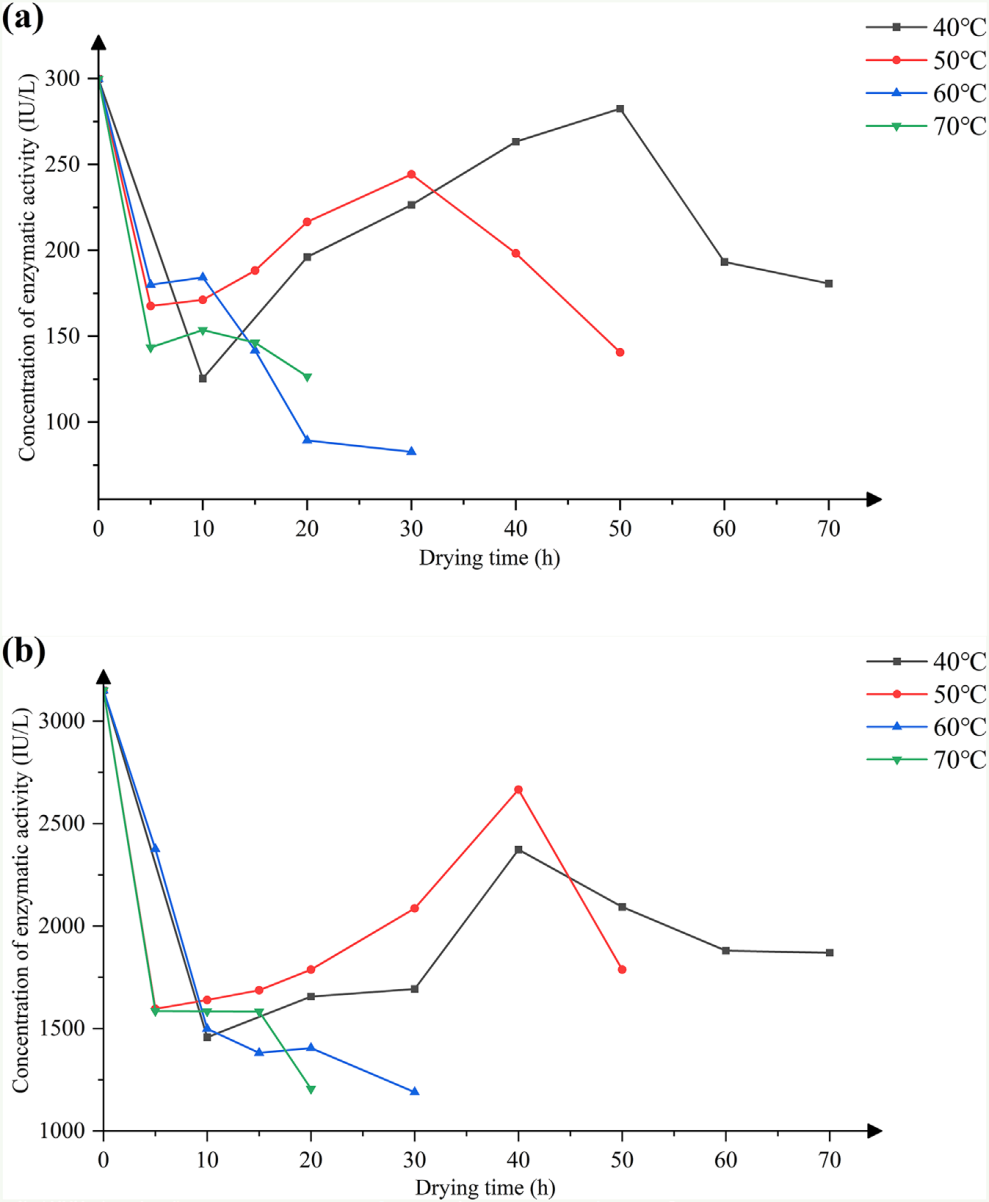
Concentrations of enzyme activity of 1,8‐cineole cyclase (a) and lipoxygenase (b) during different drying temperatures.

Flavor enzymes participate in the biosynthesis pathway of characteristic aroma components in *A. tsaoko* , forming flavor precursors and aroma components. These enzymes directly affect the flavor profile of food, enriching its flavor components and overall taste (Hadi et al. 2013). In the early stage of drying, heat stress activates the flavor enzymes, leading to enzyme catalysis based on the aroma component synthesis pathway, and accelerating the formation of volatile compounds. The activity of flavor enzymes is influenced by varying drying temperatures and time (Ashie, Simpson, and Smith 2009; LÓPez et al. 2007). The activity of two flavor enzymes in *A. tsaoko* during different drying temperatures is shown in Figure 2. During the drying process, the activity of 1,8‐cineole cyclase (1,8‐CC) shows a trend of first increasing and then decreasing. At 40°C, the enzyme activity of 1,8‐CC peaks at 50 h, significantly higher than at other time points (*p* < 0.05); at 50°C, the highest activity of 1,8‐CC is observed at 30 h (*p* < 0.05). However, at 60°C and 70°C, the enzyme activity of 1,8‐CC is activated after heating at 10 h and then showed a steady decline. The enzyme activity of lipoxygenase (LOX) also follows a similar pattern at 40°C and 50°C, peaking at 40 h under these conditions (*p* < 0.05). While under high‐temperature drying conditions (60°C and 70°C), its enzyme activity continuously decreases. The activities of both 1,8‐CC and LOX are greatly affected by high temperatures. During the later stages of drying at 60°C and 70°C, their activities decrease due to continuous heating, which may lead to enzyme denaturation and inactivation. Interestingly, these enzymes still exhibit activity activation in the later stages of drying at 40°C and 50°C, suggesting a certain degree of thermal stability at these temperatures. This indicates that 40°C and 50°C may be the optimal temperatures for these enzymes. Meanwhile, based on the analysis of enzyme activity, it is speculated that under low‐temperature drying conditions (40°C and 50°C), the enzyme catalysis can reach 30–50 h, however, after 50 h, the enzyme activity will significantly decrease, reducing the efficiency of synthesizing characteristic flavor components. Under high‐temperature drying conditions (60°C and 70°C), although there is initial activation and a brief increase in enzyme activity, indicating that flavor enzyme is a thermal activation enzyme, the overall trend of enzyme activity is decreasing; it may be inactivated due to excessive heat.

The enzyme activities during the drying process of *A. tsaoko* were lower compared to that of the fresh fruit. It is speculated that the possible reasons are as follows: first, the flavor enzyme in *A. tsaoko* is also involved in its growth and metabolism. After harvesting, the fruit's metabolism decelerates and its enzyme activity decreases. Second, the drying temperature exceeds the optimal temperature for enzyme activity and growth, leading to the production of stress factors that hinder enzyme activity due to temperature stimulation. Third, instantaneous heating can cause severe movement of water molecules, increase cell membrane permeability, and begin the aggregation of enzyme proteins to form large molecules, leading to the embedding of active centers. As the drying time increases, enzyme molecules adapt to external stress environments through their structural adjustments, thereby exposing their active centers, and leading to a surge in enzyme activity. In the later stage of drying, the enzyme molecules lose bound water, causing the enzyme to lose its activity and forcing the enzyme catalysis to stop (Li et al. 2022a). From the detection results of enzyme activity, there is indeed an activation of enzyme during the early drying stage, but different drying temperatures have different effects on the degree and timing of this enzyme activation.

### 3.3 Metabolite Analysis During Different Drying Temperatures

**FIGURE 3:**
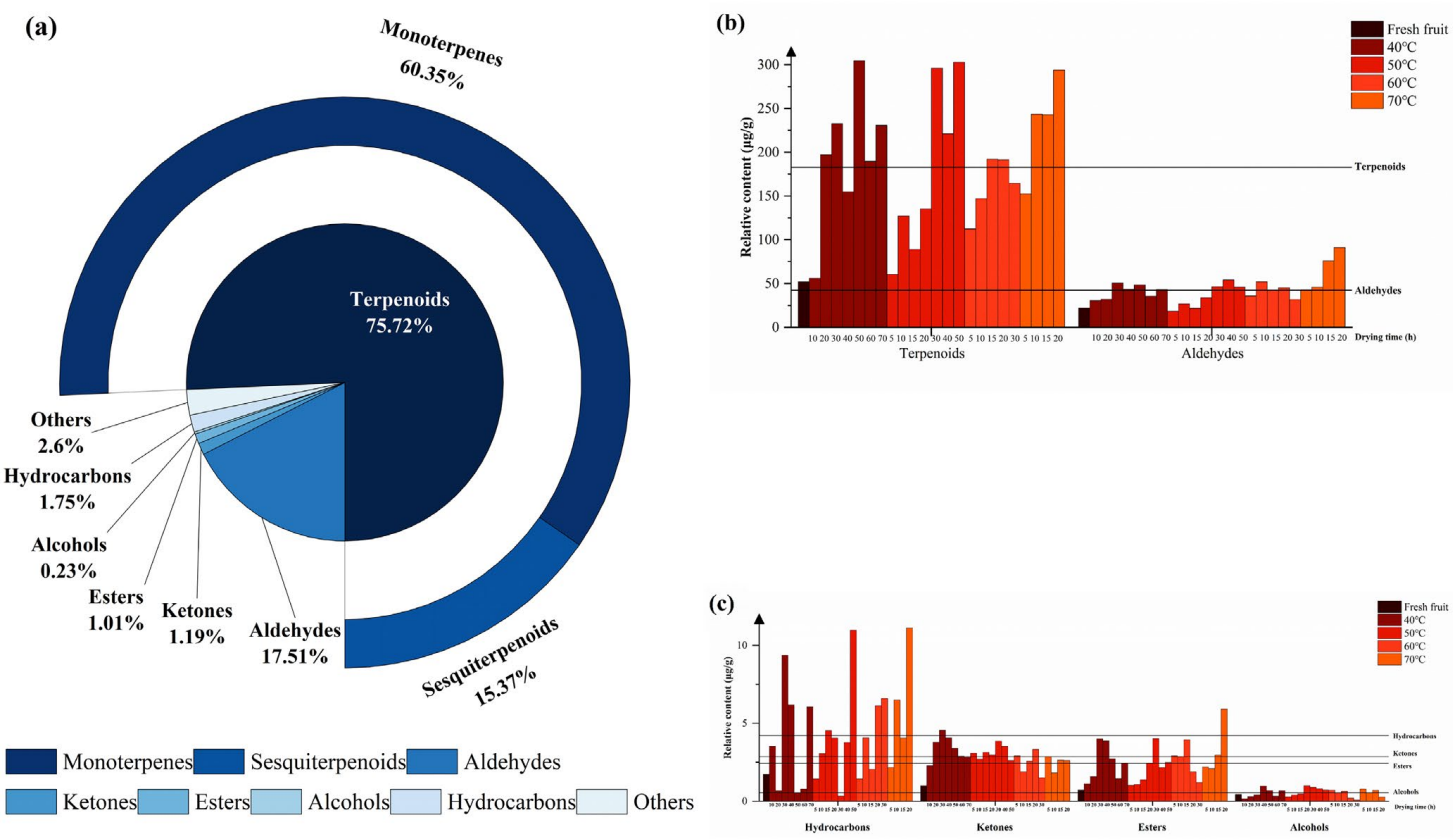
Statistical analysis of GC–MS data of *A. tsaoko* at different drying temperatures and times. (a) The proportions of relative content of various compounds in all samples; (b) the relative content of terpenoids and aldehydes; (c) the relative content of hydrocarbons, ketones, esters, and alcohols. Each horizontal line in the (b) and (c) plots corresponds to the average of the relative contents of the various compounds.

HS‐SPME coupled with gas chromatography–mass spectrometry (HS‐SPME‐GC–MS) was used to detect volatile metabolic components in *A. tsaoko* at different temperature processes. A total of 260 metabolites were detected, which can be classified into terpenoids (126 metabolites), aldehydes (39 metabolites), ketones (25 metabolites), esters (20 metabolites), alcohols (16 metabolites), hydrocarbons (13 metabolites), and others (21 metabolites). The proportions of various compounds' relative content are shown in Figure 3a. Terpenoids account for the highest proportion, reaching 75.72%, with monoterpenoids accounting for 60.35% and sesquiterpenoids accounting for 15.37%. This indicates that terpenoids are the main and important volatile metabolites in *A. tsaoko* . The relative content accounts for aldehydes (17.51%), hydrocarbons (1.75%), ketones (1.19%), esters (1.01%), alcohols (0.23%), and the rest were relatively low. The results of this study were similar to previous studies on *A. tsaoko* (Li et al. 2022b; Liao et al. 2022). Figure 3b,c shows the changes in the relative content of various compounds in *A. tsaoko* at different drying treatments. Evidently, variations in drying temperature and time significantly impact the compound composition. The relative content of compounds generally shows an increasing trend as drying time increases, which may be related to the rapid evaporation of water from the fruit during the drying process. The decrease in water in samples leads to an increase in the content of other substances. The relative content of terpenoids and aldehydes in the samples is the highest, averaging 182.9053 and 42.3047 μg/g, respectively, highlighting their importance as aroma components in *A. tsaoko* .

#### 3.3.1 Terpenoids

Terpenoids, as the main components of *A. tsaoko* , play an important role in its growth metabolism, physiological activity, and nutritional value. The relative content of all detected terpenoids is shown in Table S1. The total terpenoid content in *A. tsaoko* increases significantly with prolonged drying time. The relative content of total terpenoids in samples dried at 40°C for 50 h is the highest among different drying treatments, followed by samples dried at 50°C for 50 h. Compared to fresh fruits, the relative content of terpenoids increased from 51.9129 to 304.7205 μg/g in samples dried at 40°C for 50 h, and the relative content of terpenoids increased to 302.8897 μg/g in samples dried at 50°C for 50 h. The total terpenoid content in the samples under high‐temperature drying conditions (60°C and 70°C) is lower than that in the samples under low‐temperature drying conditions (40°C and 50°C). This indicates that high temperatures may promote the loss of major terpenoids through volatilization or degradation, thereby reducing the edible and medical value of the dried fruits. Because most terpenoids are volatile, the drying temperature is a critical determinant. From this, drying at 40°C and 50°C for 50 h is the optimal drying condition for obtaining the high terpenoid content in *A. tsaoko* .

As drying time increases, the relative content of most terpenoids shows a trend of first increasing and then decreasing. This is attributed to the loss of volatile aroma components during the later stages of drying, which is consistent with the measured essential oil content results. The high relative content of terpenoids in the samples includes cineole, (−)‐β‐pinene, (+)‐α‐pinene, geranial, α‐muurolene, terpineol, (*Z*)‐*β*‐ocimene, *γ*‐muurolene, neral, and (+)‐limonene, consisting of 8 monoterpenoids and 2 sesquiterpenoids. These are the main metabolic and pharmacologically active ingredients in *A. tsaoko* . Among them, cineole is the metabolite with the highest relative content, reaching 14.4352 μg/g in fresh fruits. In the early stage of drying (0–20 h), higher temperatures lead to a faster increase in relative content, indicating that a higher temperature in the early drying stage is conducive to the accumulation of cineole. In the later stage of drying, the relative content of cineole decreases. Among the samples meeting moisture requirements, the sample dried at 50°C has the highest relative content of cineole, which is 77.5555 μg/g.

#### 3.3.2 Aldehydes

Aldehydes are the second highest relative content compounds, playing an important role in the flavor and aroma of *A. tsaoko* . In comparison to fresh fruits, the number and relative content of aldehydes in dried fruits increase. After drying at 70°C, the number of aldehydes in the fruit increases from 26 to 38. The relative content of total aldehydes in fruits dried at 70°C is the highest, increasing from 22.2143 to 91.0710 μg/g. The relative content of aldehydes including (*E*)‐2‐decenal, 2‐dodecenal, (*E*)‐2‐dodecenal, (*E*)‐2‐ooctenal, and decaldehyde is the highest in *A. tsaoko* . During the drying process, there are significant changes in the relative content of these aldehydes, and the formation of aldehydes mainly comes from lipid oxidation degradation and the Maillard reaction (Zhou et al. 2019). These main aldehydes show a trend of increasing relative content during the drying process. Specifically, (*E*)‐2‐decenal is the aldehyde compound with an orange aroma, and it is commonly used in making spices and food additives and holds the highest relative content among fresh fruits at 12.6285 μg/g. Its relative content experiences a notable increase to 49.1121 μg/g after drying at 70°C for 20 h. The relative content of 2‐dodecenal increases the most significantly, especially when dried at 70°C for 15 h, escalating from 1.3769 to 14.3771 μg/g. It can be seen that 70°C is the optimal drying temperature for obtaining richer aldehydes and higher levels of total aldehyde content. This indicates that a higher drying temperature is conducive to lipid oxidation degradation and the Maillard reaction, thereby promoting aldehyde generation.

### 3.4 Multivariate Data Analysis of Metabolites From Differently Processed Samples

**FIGURE 4:**
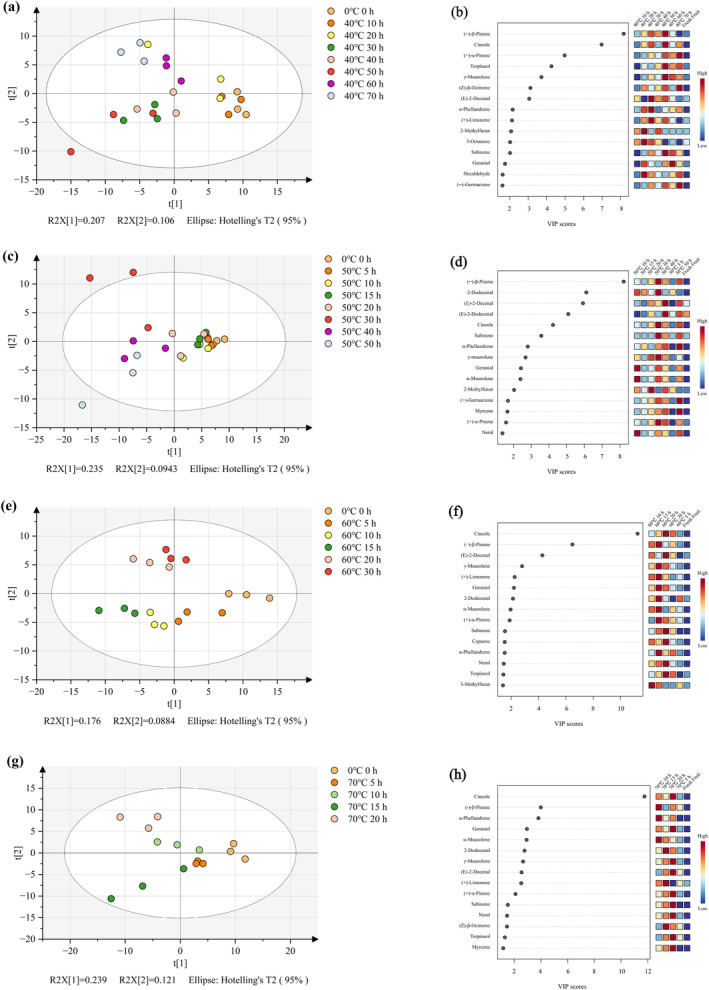
Multivariate analysis of volatile compounds using PLS‐DA during hot air drying of *A. tsaoko* . PLS‐DA score plot (a) and volatile compounds (b) ranked by VIP scores at 40°C, PLS‐DA score plot (c) and volatile compounds (d) ranked by VIP scores at 50°C, PLS‐DA score plot (e) and volatile compounds (f) ranked by VIP scores at 60°C, PLS‐DA score plot (g) and volatile compounds (h) ranked by VIP scores at 70°C.

To further intuitively explore the differences between samples and identify specific volatile compounds that lead to flavor differences in samples, partial least squares‐discriminant analysis (PLS‐DA) was used to analyze the volatile compounds. The PLS‐DA scores plot of volatile compounds under different drying temperature conditions are shown in Figure 4. In the score plot, the different samples are separated, which further indicate differences in their flavor substances. Figure 4a shows all samples are divided into three clusters at a drying temperature of 40°C. The samples dried for 0–20 h are clustered along the positive axis of PC1, while the remaining samples (30–70 h) are distributed along the negative axis of PC1. PC2 divides the sample dried for 30–70 h into two parts. The samples dried for 30–50 h are distributed on the negative axis of PC2, while the samples dried for 60–70 h are distributed on the positive axis of PC2. The PLS‐DA VIP method was used to screen important volatile compounds in different samples. The higher the VIP value, the greater the difference in the volatile compound. Figure 4b underscores the significant contributions of volatile compounds like (−)‐β‐pinene, cineole, (+)‐α‐pinene, terpineol, and γ‐muurolene to the samples' flavor profiles at 40°C. At 50°C, Figure 4c shows a similar clustering pattern, with samples dried for 0–20 h grouped on the positive side of PC1 and the rest distributed along its negative axis. PC2 further distinguishes samples dried for 30 h (positive axis) from those dried for 40–50 h (negative axis). The most influential volatile compounds at this temperature include (−)‐β‐pinene, 2‐dodecanal, (*E*)‐2‐decanal, (*E*)‐2‐dodecanal, and cineole (Figure 4d). At 60°C, Figure 4e reveals two clusters. In comparison to the samples treated at low temperatures (40°C and 50°C), the samples treated at high temperatures were completely separated in the score plot. The samples dried for 0–15 h are distributed on the negative axis of PC2, while dried for 20–30 h are on the positive axis of PC1. The volatile compounds primarily responsible for these flavor differences are cineole, (−)‐β‐pinene, (*E*)‐2‐decental, γ‐muurolene, and (+)‐limonene (Figure 4f). Finally, at 70°C, Figure 4g shows complete separation of samples, indicating pronounced flavor variations. The key volatile compounds contributing to the separation are cineole, (−)‐β‐pinene α‐phellandrene, geranal, and α‐muurolene.

### 3.5 Screening and Correlation Analysis of Differential Metabolites

**FIGURE 5:**
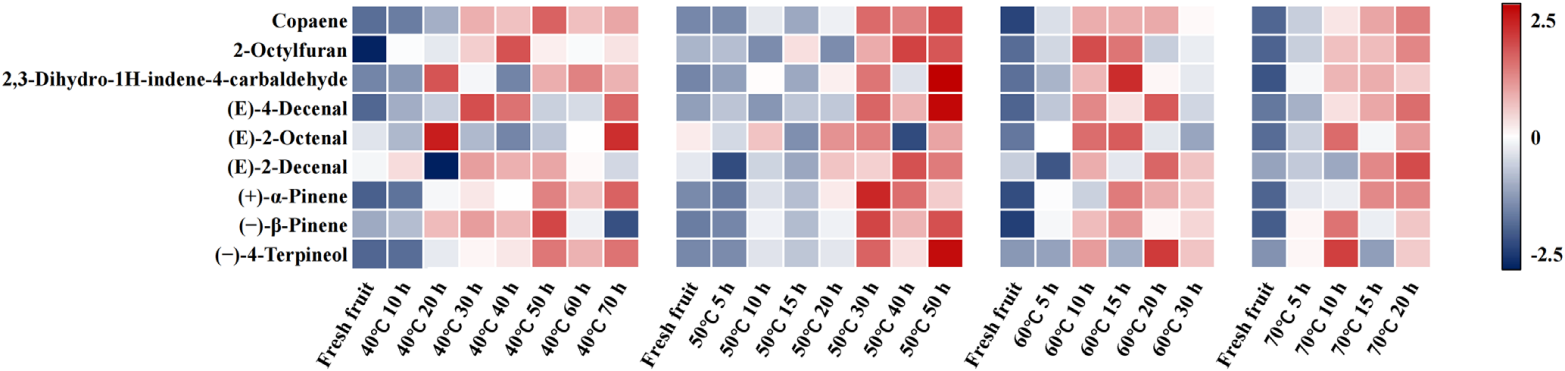
The heatmap of differential metabolites under different drying conditions.

Then, to further determine the volatile compounds that cause flavor differences during the drying of samples, a screening condition with VIP > 1 and *p* < 0.05 was used to screen out the differential metabolites. A total of nine differential metabolites were screened, including copaene, 2‐octylfuran, 2,3‐dihydro‐1 h‐indene‐4‐carbaldehyde, (*E*)‐4‐decenal, (*E*)‐2‐octenal, (*E*)‐2‐decenal, (+)‐α‐pinene, (−)‐β‐pinene, and (−)‐4‐terpineol. These nine differential metabolites can be considered as potential flavor markers for distinguishing different drying treatments of *A. tsaoko* . These metabolites are spice components crucial to the flavor of dried fruits, including 2‐octylfuran, (*E*)‐4‐decinal, (*E*)‐2‐octenal, (*E*)‐2‐decanal, (+)‐α‐pinene, (−)‐β‐pinene, (−)‐4‐terpineol, with green, fatty, turpentine, or woody aromas. Additionally, some of these compounds, such as copaene, (*E*)‐2‐octenal, (*E*)‐2‐decanal, (+)‐α‐pinene, (−)‐4‐terpineol, have insecticidal and antibacterial effects, making them important active ingredients in pharmacological effects of *A. tsaoko* . The differences in differential metabolites among samples at different drying treatments are shown in Figure 5. It can be observed that there were no significant changes in the first 10 h at 40°C and 50°C, and in the first 5 h at 60°C and 70°C. However, their relative content significantly increases after these time points. Among them, drying at 50°C for 50 h resulted in the most significant increase in the relative content of 2,3‐dihydro‐1 h‐indene‐4‐carbaldehide, (*E*)‐4‐decimal, and (−)‐4‐terpineol. Overall, based on the above metabolite analysis, it can be preliminarily concluded that the optimal drying conditions for *A. tsaoko* are drying at 50°C for 50 h.

**FIGURE 6:**
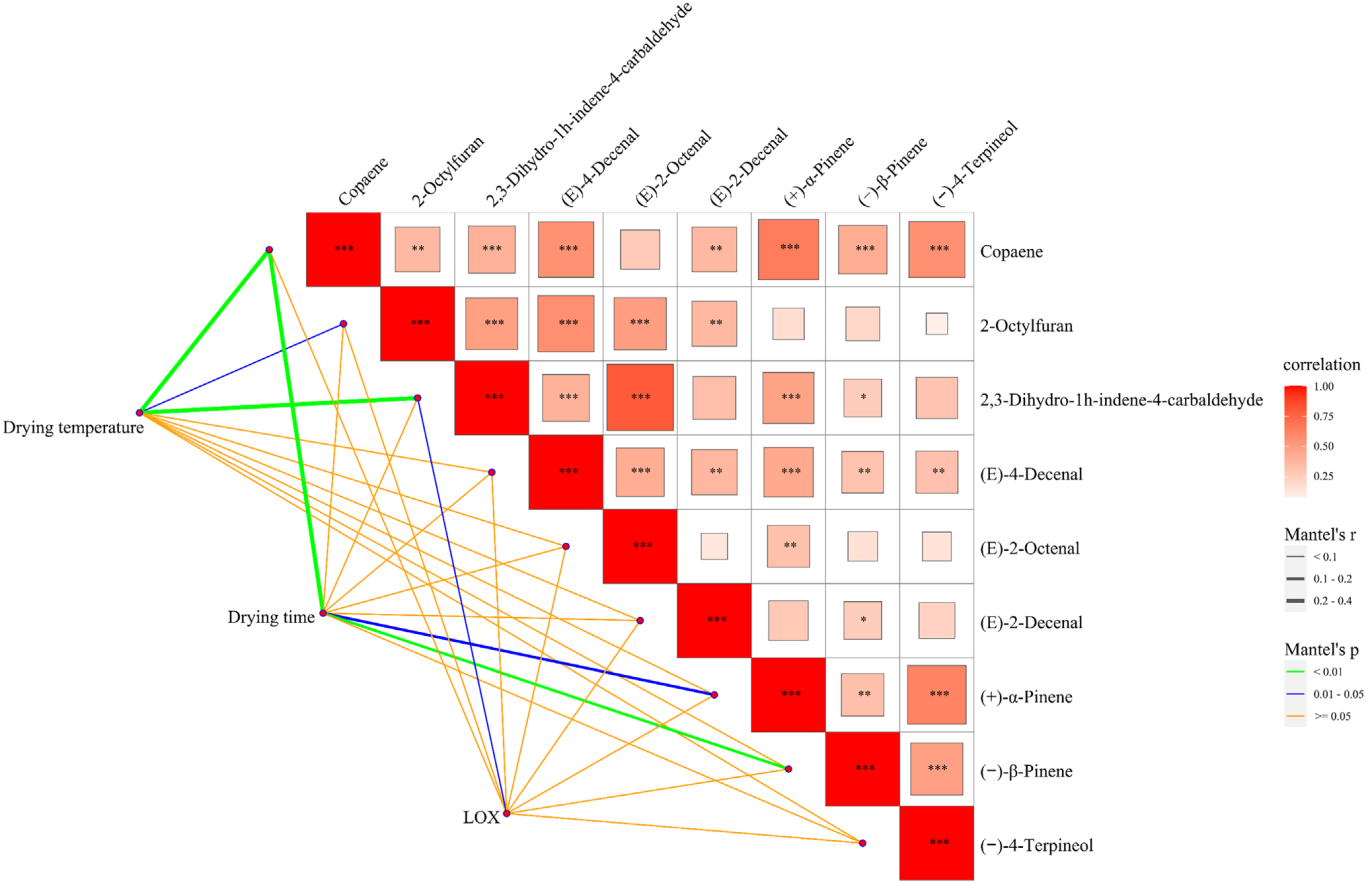
The correlation analysis between differential metabolites and drying temperature, drying time, and enzyme activity by Mantel test. The upper right graph shows the correlation heatmap of nine differential metabolites. The color gradient denotes the correlation coefficients. The bottom left graph shows the Mantel test between the influencing factors (drying temperature, drying time, and LOX enzyme activity) and the differential metabolites. LOX, lipoxygenase.

To explore the correlation between differential metabolites, a correlation heat map was established (Figure 6, upper right). In addition, the correlations between differential metabolites and drying temperature and time were established using the Mantel test (Figure 6, bottom left). The result of the correlation heat map indicated a positive correlation between the content of differential metabolites. The strongest correlation existed between (*E*)‐2‐octenal and 2,3‐dihydro‐1 h‐indene‐4‐carbaldehyde. There was also a significant positive correlation between the three terpenoids: copaene, (+)‐α‐pinene, and (−)‐4‐terpineol. The Mantel test results showed that there was a highly significant positive correlation (*p* < 0.01) between drying temperature and copaene and 2,3‐dihydro‐1 h‐indene‐4‐carbaldehyde, and a significant correlation (0.01 < *p* < 0.05) with 2‐octylfuran. Drying time had a highly significant positive correlation with copaene and (−)‐β‐pinene (*p* < 0.01), and a significant positive correlation with (+)‐α‐pinene (0.01 < *p* < 0.05). This indicates that both drying temperature and time are key influencing factors for aroma formation during the drying process of *A. tsaoko* . In addition, there was a significant correlation (0.01 < *p* < 0.05) between the activity of lipoxygenase and 2,3‐dihydro‐1 h‐indene‐4‐carbaldehyde. At present, there are few reports on 2,3‐dihydro‐1 h‐indene‐4‐carbaldehyde. This metabolite has only been detected in *A. tsaoko* , and has not been found in other species (Yang et al. 2022). Therefore, it can be used as a biomarker for *A. tsaoko* . As for the fatty aldehydes, such as (*E*)‐4‐decenal, (*E*)‐2‐octenal, and (*E*)‐2‐decenal, they can be synthesized through the oxidation of polyunsaturated fatty acids catalyzed by LOX (Zhang et al. 2023). However, there is no significant correlation between these fatty aldehydes and LOX, possibly due to the low boiling point of these fatty aldehydes causing the loss of content by the drying process exceeding the content of enzyme catalyzed synthesis. Based on the comprehensive correlation values, the degree of influence on the aroma components of *A. tsaoko* during the drying process is as follows: drying temperature > drying time. This indicates that drying temperature is the most important influencing factor in the drying process. Therefore, in actual production, attention should be paid to controlling the drying temperature in order to obtain high‐quality dried fruit.

Currently, the primary research directions of *A. tsaoko* encompass quality control and pharmacological effects. On the one hand, essential oils serve as a distinctive metric for assessing the quality of *A. tsaoko* , particularly 1, 8‐cineole. However, their ubiquitous presence in plant essential oils renders them unsuitable as a “Q‐marker” for *A. tsaoko* (Pu et al. 2022). The nine differential metabolites identified in this study can be considered potential candidates for the “Q‐marker” of dried fruit, thereby offering a theoretical foundation for establishing quality evaluation standards for *A. tsaoko* . On the other hand, despite *A. tsaoko* possessing multiple potential pharmacological activities, a deeper understanding of the regulatory molecular mechanisms of its key compounds is necessary (He, Yang, and Wang 2023). *A. tsaoko* are used as medicine and food as dried fruits, so it is crucial to focus on the metabolites present in dried fruits. This study provides a preliminary foundation for further research into the pharmacological effects and molecular mechanisms of individual compounds within *A. tsaoko* .

## 4 Conclusions

In this study, significant changes were observed in the flavor morphology and composition of *A. tsaoko* during the drying process. The morphological changes were most significant in the early stage of drying. The oil yield of *A. tsaoko* initially increased with drying time but then decreased, and excessively long drying periods could result in lower oil yields. In the early stage of drying, enzymes were another influencing factor of metabolite changes. In the later stage of drying, enzymes gradually became inactive and metabolite changes tend to stabilize. A total of 260 metabolites were detected by gas chromatography–mass spectrometry, with terpenoids and aldehydes as the main metabolites. Copaene, 2‐octylfuran, 2,3‐dihydro‐1 h‐indene‐4‐carbaldehyde, (*E*)‐4‐decenal, (*E*)‐2‐octenal, (*E*)‐2‐decenal, (+)‐α‐pinene, (−)‐β‐pinene, and (−)‐4‐terpineol were screened as differential metabolites, can be considered as potential flavor markers for distinguishing different drying treatments of *A. tsaoko* . Comprehensive analysis of morphology, oil yield, moisture content, and metabolite revealed that drying temperature is the most important influencing factor, and the optimal drying condition for *A. tsaoko* is dried at 50°C for 50 h. Therefore, by controlling the drying conditions, the flavor quality of dried fruits of *A. tsaoko* can be effectively improved. This study provides theoretical guidance for the processing and production of *A. tsaoko* .

## Author Contributions

**Hui Wen:** data curation (equal), formal analysis (equal), writing – original draft (equal). **Meiquan Yang:** investigation (equal), supervision (equal), validation (equal). **Zongliang Xu:** investigation (equal), project administration (equal), resources (equal). **Tianmei Yang:** conceptualization (equal), methodology (equal), supervision (equal). **Jinyu Zhang:** conceptualization (equal), funding acquisition (equal), writing – review and editing (equal).

## Conflicts of Interest

The authors declare no conflicts of interest.

## Supporting information


**Data S1.** Supporting Information.

## Data Availability

The authors have nothing to report.
